# Diet Quality Is Associated with Serum Antioxidant Capacity in Women with Breast Cancer: A Cross Sectional Study

**DOI:** 10.3390/nu13010115

**Published:** 2020-12-30

**Authors:** Luiza K. Reitz, Sheyla de L. Baptista, Elaine da S. Santos, Patrícia F. Hinnig, Gabriele Rockenbach, Francilene G. K. Vieira, Maria A. A. de Assis, Edson L. da Silva, Brunna C. B. Boaventura, Patrícia F. Di Pietro

**Affiliations:** 1 Post Graduate Program in Nutrition, Health Sciences Center, Federal University of Santa Catarina, Florianopolis 88010790, SC, Brazil; luizakreitz@gmail.com (L.K.R.); sheyladeliz@gmail.com (S.d.L.B.); nutricionistaelaine@hotmail.com (E.d.S.S.); phinnig@yahoo.com (P.F.H.); francilene.vieira@ufsc.br (F.G.K.V.); maria.assis@ufsc.br (M.A.A.d.A.); dasilvael@hotmail.com (E.L.d.S.); brunnab@gmail.com (B.C.B.B.); 2 Department of Nutrition, Health Sciences Center, Federal University of Santa Catarina, Florianopolis 88010790, SC, Brazil; gabrielerockenbach@gmail.com; 3 Department of Clinical Analyses, Health Sciences Center, Federal University of Santa Catarina, Florianopolis 88010790, SC, Brazil

**Keywords:** oxidative stress, diet quality, breast cancer, adjuvant treatment

## Abstract

Oxidative stress produced by adjuvant treatments is associated with cell injury; however, a healthy diet can help mitigate it. The aim of this study is to investigate the association between diet quality and oxidative stress parameters in women subjected to adjuvant treatment for breast cancer. The sample comprised 70 women. Oxidative stress biomarkers and diet quality parameters based on the Brazilian Healthy Eating Index—Revised (BHEI-R)—were evaluated at baseline (p0) and after adjuvant treatment (p1). Ferric reducing antioxidant potential (FRAP) was associated with diet quality at p0. BHEI-R scores were not different between p0 and p1; however, scores from total vegetables, total fruits, milk and dairy products, and meat, eggs and legumes were lower during treatment. On the other hand, lower sodium and saturated fat intake observed at p1 counterbalanced the BHEI-R score. Oxidative stress parameters have increased at p1, but they were not associated with diet quality; thus, changes in component intake were not enough to promote changes in oxidative stress during treatment. It appears that diet can enhance patients’ antioxidant defense before treatment, which could lead to better outcomes in the long term. Further investigations may help to clarify the association between diet and oxidative stress in women with breast cancer.

## 1. Introduction

Breast cancer is the most common type of cancer and the leading cause of cancer-related death among women worldwide [1,2,3,4]. According to estimates, 66,280 new breast cancer cases are expected to be diagnosed among women in Brazil in 2020 [5].

Evidence has suggested that reactive oxygen species (ROS) may be involved in the onset, promotion and progression of breast cancer stages [6]. Adjuvant chemotherapy and radiotherapy treatments can increase the oxidative stress produced by cancerous cells due to ROS production, whose cytotoxic effect leads to DNA damage capable of killing tumor cells and stopping their proliferation [7,8,9,10]. Chemotherapy drugs are associated with high lipid peroxide and protein carbonyl levels in comparison to non-treatment. This finding indicates that these therapies are associated with increased oxidant status [8]. However, chemotherapy treatments comprise high drug doses capable of killing both cancerous and healthy cells [11]. Therefore, improving treatment efficacy and reducing side effects is of paramount importance for public health.

Previous studies have suggested that healthy diets comprising several types of vegetables, edible plants and fruits can be beneficial for cancer patients, since they can help stop disease progression due to bioactive compounds found in these diets and their antioxidant, anti-inflammatory and anti-proliferative effects [12,13]. The intake of vegetables, mainly with green leaves, and fruits has shown an inverse association with genomic instability, which is associated with oxidative stress, reduces tumor cell replicative and proliferative activities, and improves side effects of chemotherapy and radiotherapy on healthy cells [14].

Diet quality indices provide information about the dietary patterns of individuals or populations. They reflect total diet rather than using a single food-intake determination method, such as diet component or nutrient [15]. Meta-analysis applied to seven observational studies comprising 319,993 participants showed an inverse association between better diet quality measured through dietary inflammatory index and risk of developing breast cancer [16]. Moreover, previous studies have shown that better dietary indices were associated with better prognosis [17] and with a lower risk of dying from breast cancer [18,19].

Although dietary indices have been used to access the quality of diets adopted by women with breast cancer [20,21], the association between these diets and oxidative stress biomarkers remains unclear, mainly if one takes into consideration the period before and after adjuvant disease treatment.

Given the important role played by diet quality during chemotherapy and radiotherapy treatment for breast cancer, the aim of the current cross-sectional study was to evaluate the association between diet quality and oxidative stress biomarkers in women subjected to adjuvant treatment for breast cancer. The hypothesis of the present study is that diet quality can help to reduce oxidative stress biomarker levels in women subjected to adjuvant treatment for breast cancer by likely having a beneficial effect on them in the long term.

## 2. Materials and Methods

### 2.1. Study Design and Sampling

A cross-sectional study was performed with a convenience sample comprising women who underwent surgical treatment for breast cancer at Carmela Dutra Hospital from 2006 to 2011. Subsequently, these women were subjected to adjuvant treatment at the Cancer Research Center (CEPON), in Florianopolis City, Santa Catarina State, Brazil.

**Figure 1:**
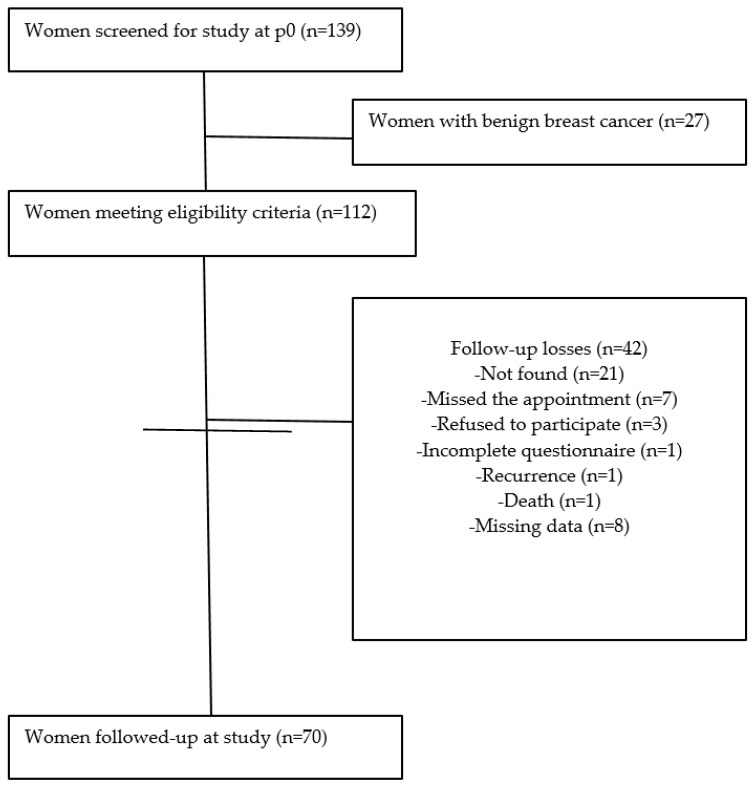
Flowchart of participant selection at baseline and follow-up. Florianopolis City, Santa Catarina State, Brazil.

Eligibility criteria considered all women admitted to the hospital for surgical treatment of breast cancer who were under suspicion of having a malignant tumor or who were diagnosed with breast cancer. Malignancy suspicion was confirmed in the anatomopathological report issued after surgery. Exclusion criteria comprised the history of cancer or previous surgical procedure in a period equal to, or shorter than, one year; confirmation of benign tumors without malignancy suspicion; pregnant and lactating women; patients who tested positive for human immunodeficiency virus; and individuals who underwent neoadjuvant antineoplastic treatment [22]. Initially, 139 women were recruited. After evaluation based on the eligibility criteria, 70 women who had finished treatment, presented complete data and had signed the written consent were included in the study (Figure 1).

Data were collected at two different times: at breast cancer diagnosis (p0), and after the end of cancer treatment (p1). Data collection at p0 took place on the same day the anatomopathological examination was performed (diagnosis). Participants’ mean cancer treatment time was 17.9 ± 6.2 months; p1 data were collected 8.9 ± 3.4 months after the end of cancer treatment.

The present study was carried out in full compliance with the Declaration of Helsinki and written in accordance with the Strengthening the Reporting of Observational Studies in Epidemiology (STROBE) statement for reporting cross-sectional studies. It was approved on 30 May 2008 by the Human Research Ethics Committee of Federal University of Santa Catarina, by the Ethics Committee of Carmela Dutra Hospital and by the Cancer Research Center (CEPON) Ethics Committee (process number 099/08).

### 2.2. Oxidative Stress Analysis

Oxidative stress biomarkers were measured through the collection of 15 milliliters of blood (by venipuncture in the forearm) into tubes added with EDTA, or not, in order to obtain plasma and serum, respectively, through centrifugation (1000× *g*/10 min). Immediately after venipuncture, a whole blood aliquot was used to measure reduced glutathione (GSH) by using 20% trichloroacetic acid, based on the method described by Beutler et al. [23].

Lipid peroxidation was assessed based on the determination of substances reactive to thiobarbituric acid (TBARS) [24], as well as on lipid hydroperoxide (LH) levels, as suggested by Nourooz-Zadeh et al. [25], based on the oxidation of iron found in FOX2 solution. Plasma protein oxidation was determined through carbonyl levels, as described by Levine et al. [26]. Ferric reducing antioxidant potential (FRAP) assay was used to check serum antioxidant capacity, based on Benzie and Strain [27].

LH, TBARS and serum antioxidant capacity measurements were taken on the same day as the blood sample collection. A plasma aliquot was stored at −80 °C for no longer than 30 days, for further protein carbonyl determination. All biochemical analyses were performed in duplicate.

### 2.3. Diet Quality Assessment

Food intake data were collected through the Food Frequency Questionnaire (FFQ), adapted from a previously validated FFQ [28]. The aforementioned FFQ was applied by trained researchers at p0 (baseline period) and p1 (period after the end of treatment), which corresponded to the previous year, i.e., period of adjuvant treatment).

Data about individual food intake available in the FFQ were transformed into quantitative nutrient information [29], in Microsoft Excel^®^ software by using the Brazilian Food Composition Database [30] and the Food Composition Database of the United States Department of Agriculture [31].

Diet quality was evaluated based on the Brazilian Healthy Eating Index—Revised (BHEI-R) [32]. In order to do so, FFQ items were grouped according to BHEI-R components. BHEI-R was validated in Brazil [33] by taking into consideration the Brazilian Food Guidelines [34]. It is based on the following components: total fruits (including fruits and natural fruit juices); whole fruits; total vegetables; dark green and orange vegetables and legumes; total grains (including grains, roots and tubers); whole grains; milk and dairy products (including soy drinks); meat, eggs and legumes; oils (including mono and polyunsaturated fats, nuts, seeds and fish oils); saturated fats; sodium; and calories from solid fats, alcohol and added sugars—SoFAAS. Legumes were included in components “total vegetables”, and “dark green and orange vegetables and legumes” only after reaching the maximum score for “meat, eggs and legumes” [32].

The number of servings of each food group component from the BHEI-R was expressed by energy density, as well as by sodium (number of servings or mg of sodium/1000 kcal); saturated fats and SoFAAS intake was expressed as total energy intake rate [32].

The BHEI-R uses a scoring system to evaluate a set of food items. Total fruits, whole fruits, total vegetables, dark green and orange vegetables and legumes, total grains and whole grains vary from 0 to 5 points. Groups such as milk and dairy products; meat, eggs and legumes; and oils range from 0 to 10 points. Lack of intake of these groups corresponds to score zero, and the proportional calculation was computed to score the intermediate consumption. The score attributed to components such as saturated fat and sodium ranges from 0 to 10 points and is inversely proportional to their intake. The score attributed to SoFAAS ranges from 0 to 20 points and is also inversely related to its intake. The sum of scores from all components results in the final diet quality score, which can range from 0 to 100 points. The ideal overall BHEI-R score of 100 indicates that the set of food items agrees with key dietary recommendations [32]. The BHEI-R scores were converted into tertiles for analysis purposes.

### 2.4. Other Assessments

A questionnaire developed by Di Pietro et al. [35] and adapted by Vieira et al. [22] was applied by a trained interviewer. It addressed the following aspects: patients’ identification, clinical and reproductive history, socioeconomic and anthropometric information and data about the disease and treatment.

Participants’ weight, height and waist circumference (WC) were measured by trained researchers, based on standard techniques [36]. Anthropometric measurements were taken while participants were wearing light clothes and no shoes. Weight was measured in a 180-kg capacity digital scale (Marte^®^, model PP, 50 g precision). Height was measured with a stadiometer (Alturexata^®^, 1 mm precision). Body mass index (BMI) was computed as weight (in kg) divided by the square of height (in m). Weight status was evaluated based on BMI cut-off points proposed by the World Health Organization [37]. Waist Circumference (WC) was classified according to WHO cut-off points [38]. Physical Activity Level (PAL) was calculated based on the ratio between total estimated energy expenditure and basal energy expenditure; it was categorized according to the Institute of Medicine [39]. Data used to assess PAL were self-reported in the questionnaire and calculated based on the frequency and time participants expended exercising.

### 2.5. Statistical Analysis

Data were entered in Microsoft Office Excel^®^, and statistical analysis was performed in the STATA^®^13.0 software (StataCorp LP, College Station, TX, USA). The Shapiro–Wilk test was used to test data normality. Continuous variables were described as mean and standard deviation (SD), or as median and interquartile range, depending on data distribution normality. Categorical variables were described as absolute and relative frequencies.

ANOVA and Kruskal–Wallis test were used to assess associations between continuous variables and BHEI-R scores in tertiles; they were followed by Bonferroni or Mann–Whitney posthoc tests. Trend analysis (ANOVA or nonparametric trend test) was used when a trend was observed in data. The Chi-squared test was used to associate qualitative variables with BHEI-R scores in tertiles. Paired t or Wilcoxon tests were performed, according to data normality, to access differences in continuous variables between p0 and p1.

Variables such as TBARS, carbonyl and LH were transformed into logarithm and multiple linear regressions and used to investigate the association between diet quality and oxidative stress parameters at p0 and p1. Simple linear regression of each variable and outcome was initially performed. Confounder variables such as alcohol intake, tumor stage, schooling, surgery type, tumor classification, waist circumference, PAL, race and age were included as independent variables in the regression analysis.

Logistic regression was used to investigate whether changes in diet quality score were associated with changes in oxidative stress parameters. Variable “changes in diet quality score” (defined as the difference between diet quality score before and after treatment) was dichotomized into “increase” or “decrease” in diet quality score after treatment. The same process was applied to changes in oxidative stress parameters, which were dichotomized into an increase or decrease in oxidative stress parameters after treatment. Variables such as surgery type, difference in waist circumference, tumor stage, alcohol intake and age were also tested as independent variables to evaluate potential confounders. Univariate logistic regression was initially performed and resulted in crude odds ratio (OR) values.

Variables presenting statistically significant regression coefficient values at *p* ≤ 0.2 were selected for the final model, in both regression models, except for age, which remained in all adjustment models. Statistical significance level was set at 5%.

Given the absence of studies focused on investigating the association between diet quality and oxidative stress in women with breast cancer, the current study used results of the correlation of vegetable intake and saturated fat energy rate to oxidative stress biomarkers (Total Antioxidant Status)—i.e., 0.42 and −0.38, respectively—recorded for patients with rheumatoid arthritis [40]. Type I error of 0.05 and study power of 80% were taken into consideration for sample calculation, based on the formula described by Bowner et al. [41].

## 3. Results

The total sample comprised 70 women (missing oxidative stress data at p0 = 3 and at p1 = 7), at mean age of 52.2 years (SD = 10.7 years). With respect to participants’ features, 64.2% (*n* = 45) were overweight or obese, 92.8% (*n* = 65) were diagnosed with invasive carcinoma and 64.2% (*n* = 45) were classified at tumor stages II and III. Most patients were subjected to radiotherapy in association with chemotherapy (42.8%, *n* = 30) and with tamoxifen used as hormonal therapy (72.8%, *n* = 51). Sociodemographic, clinical and anthropometric data based on BHEI-R tertiles recorded at the baseline period (p0) are shown in Table 1. Only age has shown statistical significance in the BHEI-R index; the highest mean BHEI-R index was recorded in the 3rd score tertile of diet quality. There were no differences between other features of the analyzed women and diet quality tertiles at baseline (p0) (Table 1).

Table 2 shows the comparison of BHEI-R index score, components and oxidative stress biomarkers between p0 and p1. Although the median BHEI-R index score was not different between p0 and p1, food group scores recorded for total vegetables, milk and dairy products, and meat, eggs and legumes were significantly lower at p1, and it indicated reduced intake of these food groups during treatment. On the other hand, median BHEI-R scores recorded for whole fruit, sodium and saturated fat were significantly higher at p1 (Table 2). Median values recorded for oxidative stress parameters such as TBARS, LH, and carbonyl were higher after treatment (*p* < 0.001, *p* = 0.046, and *p* = 0.023, respectively).

The multiple linear regression model has shown a positive association between FRAP and higher tertile BHEI-R index score after adjustment for alcohol intake, schooling and age-only at baseline (*p* = 0.019) (Table 3). Logistic regression did not show associations between diet quality score and the analyzed oxidative stress biomarkers (Table 4).

## 4. Discussion

The present study has investigated the association between diet quality and oxidative stress biomarkers, before and after adjuvant treatment, in women diagnosed with breast cancer. Important differences in some individual food group scores were found between p0 and p1, although it was not possible to find variation in global BHEI-R index score. In addition, antioxidant biomarker FRAP was associated with higher diet quality index score at baseline. Previous studies had investigated FRAP in women with breast cancer [42,43,44]. Evidence has shown that FRAP values were proportional to the reducing power of the main nonenzymatic antioxidants in plasma [27]; thus, this assay was selected to assess the antioxidant status of women with breast cancer in the present study. Sateesh et al. [42] found values of FRAP in women with breast cancer lower than the ones recorded for healthy women; this finding indicates that the disease leads to decreased nonenzymatic antioxidant defenses. Singh et al. [44] found lower FRAP values after neoadjuvant chemotherapy application in women with breast cancer, which evidenced an even greater reduction in nonenzymatic antioxidant defense caused by the treatment. According to the present investigation, it seems that patients’ diet can enhance the nonenzymatic antioxidant defenses reduced by the disease, and it likely has a protective effect against oxidative damage in the case of lack of treatment. Thus, a better-quality diet based on the intake of vitamins, minerals and bioactive compounds can help improve patients’ antioxidant defense and may be associated with better outcomes during treatment [45]. However, FRAP biomarker did not remain significantly associated with diet quality after the end of cancer treatment. This finding can be explained by exacerbated oxidative stress caused by the adjuvant treatment, since favorable changes in dietary intake were not enough to overcome it, thereby not affecting its purpose. In fact, it was also observed that oxidative stress biomarkers of lipid and protein oxidation—measured based on TBARS, LH and carbonyl—were significantly higher after the end of treatment than at baseline. In agreement with the present investigation, Galvan et al. [8] observed that 66 women with breast cancer, who were subjected to adjuvant chemotherapy, showed increased LH, carbonyl and TBARS levels after treatment. Rockenbach et al. [46] also found an increase in the same oxidative stress parameters in 40 women diagnosed with breast cancer, after adjuvant treatment with chemotherapy and/or radiotherapy. These previous studies were conducted by the same research group as the present investigation and showed similar results, which can be explained by treatment-induced ROS production to kill cancerous cells [7,8,10]. These cells, in turn, were not affected by diet quality. To date, the literature lacks publications focused on investigating the association between diet quality and oxidative stress in women subjected to adjuvant treatment for breast cancer.

It is suggested that ROS are involved in the onset, promotion and progression of carcinogenesis processes [6], since they lead to damage in intracellular macromolecules such as lipids and proteins [47]; this process leads to changes in their function [48] and generates oxidation products such as TBARS, LH and protein carbonyl, respectively [24,25,26]. Oxidative stress products have been associated with breast carcinogenesis. Lipid peroxidation is a carcinogenic factor for breast cancer [49,50]. Studies have shown that women diagnosed with breast cancer presented higher plasma protein carbonyl concentrations than healthy women, and this suggests an association with the risk of developing the disease [51,52,53,54,55], as well as with malignancy [54]. Although the present investigation did not find an association between diet and oxidative stress biomarkers in women with breast cancer, this association had been previously explored. Rockenbach et al. [46] investigated 55 women with breast cancer and showed that chicken and high-fat dairy product intake were associated with LH concentrations, despite the intake of vitamin E, which was inversely associated with it; oil intake was associated with TBARS, whereas animal fat, dairy product and sweets intake showed an inverse association with FRAP. Yeon et al. [55] have shown an inverse association between DNA oxidative damage measured by 8-OH-dG and vitamin A and b-carotene intake in women with breast cancer. An investigation conducted with women previously treated for breast cancer showed an inverse association between vitamin E intake and urinary levels of 8-OH-dG and 8-epi-prostaglandin F2α; 8-OH-dG was positively associated with arachidonic acid, which authors attributed to meat intake [56]. According to Wirth et al. [57], women with breast cancer who fed on cruciferous vegetables (≥14 cups/week) for 3 weeks showed lower urinary concentrations of 8-OH-dG after the intervention than the control group. The association between diet and oxidative stress in women with breast cancer remains unclear; thus, further investigation should be conducted.

In addition to reduced vegetable intake during cancer treatment, women investigated in the current study showed a global lower intake of milk and dairy products, as well as of meat, eggs and legumes. As expected, these changes may be linked to chemotherapy-related side effects such as lower appetite, lower self-reported taste, dry mouth and nausea [58], which can result in aversion to some food groups, such as meat [58,59,60,61] and dairy products [61]. These dietary changes can lead to lower protein intake. On the other hand, they may also be associated with lower saturated fats and sodium intake, providing higher scores in these components, counterbalancing the global BHEI-R index and leading to the absence of changes in diet quality at p1, as observed in the present study.

Although total BHEI-R score remained unchanged during treatment, which is in agreement with previous studies [20,21], reduced vegetable intake led to lower antioxidant compound intake and may be associated with poorer prognosis and higher risk of breast cancer recurrence [62,63]. Lower vegetable intake can lead to reduced fiber intake, which is negative since higher intake of this dietary component before and after diagnosis is linked to a lower risk of dying from breast cancer [64]. However, reduced meat, egg, milk and dairy product intake, which might be associated with chemotherapy-related side effects, may have led to reduced intake of saturated fats and might be considered a positive outcome after treatment, given the existing link between survival after breast cancer and lower saturated fat intake [64]. The negative outcome associated with lower vegetable intake was counterbalanced by the positive outcome associated with lower saturated fats and sodium intake. In addition, it did not change diet quality score, although it may have led to reduced antioxidant intake.

Despite the important role played by diet quality during and after breast cancer treatment in reducing the risk of disease recurrence, hypertension is the most common comorbidity in cancer patients, regardless of cancer type, and it plays important role in the prognosis and survival of women with breast cancer [65,66,67]. Reduced sodium intake during, and after, adjuvant treatment for breast cancer in the current study may be associated with a reduced intake of food items like sausages and salty cheeses, since they were taken into consideration at the time to calculate components such as milk and dairy products, as well as meat, eggs, and legumes, in the BHEI-R index. These results seem to be favorable and are in compliance with recommendations by the World Cancer Research Fund International to reduce meat and sausage intake for breast cancer prevention purposes [64], as well as to control blood pressure and, consequently, the risk of experiencing cardiovascular disease and events. In addition, salty food intake as part of an “unhealthy” food intake pattern can be associated with an increased risk of developing breast cancer [68].

The current study presented some limitations; the sample comprised women who underwent different adjuvant treatments with heterogeneous durations, and it may have potentially changed oxidative stress biomarkers at p1; food intake assessment based on FFQ may have been biased, since this method does not include a variety of food items capable of affecting diet quality assessment. In addition, the BHEI-R index does not take into consideration soy and fiber intake, which are inversely associated with death due to breast cancer after diagnosis [64]; total energy intake, since women who consumed high-energy-density diets presented higher risk of developing breast cancer [69]; dietary antioxidant intake value, which can be inversely associated with risk of developing breast cancer [70]; and salt addition to meals, which can underestimate the sodium intake. Finally, another limitation of the current study lies in the classification of changes in diet quality score as increased or decreased (Table 4), which may have influenced the non-association observed between diet quality and oxidative stress markers. Women with a remarkably high index may have a slight reduction, and still maintain a very high-quality diet, whereas the remarkably low score may slightly improve but remain very low. However, the same results were observed in the logistic regression analysis applied to variable diet quality (*p* > 0.05, data not shown). Among the strengths of the study, one finds the BHEI-R index, which evaluates the reality of the diet followed by these women based on a global diet quality assessment and may better reflect the synergic effect of different food compounds on oxidative stress parameters, rather than basing the investigation on a single diet item or bioactive compound.

## 5. Conclusions

The current study was the first to investigate the association between global diet quality parameter and oxidative stress biomarkers in women before and after adjuvant treatment for breast cancer. It has shown that the antioxidant biomarker FRAP was associated with higher diet quality index score at baseline, which could indicate the beneficial effect of the diet on the health of these women in the long-term. Although the current study did not find a difference in the global BHEI-R index score, it was possible to see some differences in scores between individual food groups, such as decreased total vegetables, milk and dairy products, and meat, eggs and legumes, with a concomitant score increase in whole fruit, sodium and saturated fat. Reduced vegetable intake during adjuvant treatment for breast cancer was a negative outcome, which may have been counterbalanced by lower sodium and saturated fats intake, which was a positive outcome. The counterbalance of diet quality score did not result from increased antioxidant-rich food intake, which could provide benefits to patients during adjuvant treatment for breast cancer. This fact can explain the lack of association with changes in oxidative stress parameters. Therefore, future investigations should adopt dietary antioxidant capacity assessment methods to investigate the association between biochemical markers of oxidative stress and antioxidant status. In addition, investigations could be based on nutritional interventions during adjuvant treatment, which may help to explain the association between dietary aspects and oxidative stress biomarkers.

## Figures and Tables

**Table 1:** Sociodemographic, anthropometric, clinical and therapeutic features of women with breast cancer, based on tertiles of the Brazilian Healthy Eating Index—Revised (BHEI-R) at baseline (P0) (*n* = 70), Florianopolis City (SC), 2006–2011.

		BHEI-R Tertiles (p0)		*p*
	1st tertile	2nd tertile	3rd tertile	
Age ^a^ (years)	48.54 (10.54)	51.96 (9.44)	56.35 (11.16)	**0.012** *
Weight ^b^ (kg)	68.00 (57.75, 81.50)	69.50 (59.60, 78.80)	65.90 (56.50, 73.00)	0.470 **
Waist circumference (cm)	82.25 (76.25, 98.50)	89.50 (83.00, 98.00)	84.0 (81.50, 95.00)	0.301 **
Smoking, *n* (%)				0.745 ^#^
Yes	6 (25.0)	4 (17.4)	6 (26.1)	
No	18 (75.0)	19 (82.6)	17 (73.9)	
Alcohol, *n* (%)				0.793 ^#^
Yes	2 (8.3)	1 (4.4)	1 (4.3)	
No	22 (91.7)	22 (95.6)	22 (95.7)	
Race, *n* (%)				0.354 ^#^
White	22 (91.7)	21 (91.3)	23 (100.0)	
Brown	2 (8.3)	2 (8.7)	0 (0.0)	
Education				0.077 ^#^
<8 years	17 (70.8)	15 (65.2)	17 (73.9)	
9–11 years	6 (25.0)	3 (13.1)	6 (26.1)	
>12 years	1 (4.2)	5 (21.7)	0 (0.0)	
BMI (kg/m^2^), *n* (%)				0.208 ^#^
<24.9 kg/m^2^	11 (45.8)	5 (21.7)	9 (39.1)	
≥25 kg/m^2^	13 (54.2)	18 (78.3)	14 (60.9)	
Tumor classification, *n* (%)				0.236 ^#^
Invasive carcinoma	21 (32.3)	21 (32.3)	23 (35.4)	
Carcinoma in situ	3 (60.0)	2 (40.0)	0 (0.0)	
Tumor stage, *n* (%)				0.604 ^#^
0	0 (0.0)	2 (8.7)	0 (0.0)	
I	9 (37.5)	7 (30.4)	7 (30.4)	
II	10 (41.7)	9 (39.1)	10 (43.5)	
III	5 (20.8)	5 (21.7)	6 (26.1)	
Treatment type, *n* (%)				0.622 ^#^
Radiotherapy	6 (37.5)	5 (31.2)	5 (31.3)	
Chemotherapy	6 (31.6)	5 (26.3)	8 (42.1)	
Radiotherapy in association with chemotherapy	12 (40.0)	10 (33.3)	8 (26.7)	
No	0 (0.0)	3 (60.0)	2 (40.0)	
Hormone therapy, *n* (%)				0.138 ^#^
Tamoxifen	21 (87.6)	15 (65.2)	15 (65.2)	
Aromatase inhibitor	3 (12.5)	8 (34.8)	8 (34.8)	
Physical Activity Level (PAL) ^b^	1.36 (1.30–1.40)	1.32 (1.30–1.34)	1.33 (1.29–1.36)	0.520 **
Surgery type, *n* (%)				0.576 ^#^
Partial mastectomy	9 (37.5)	4 (17.4)	5 (21.7)	
Radical mastectomy	10 (41.7)	13 (56.5)	13 (56.5)	
Sectorectomy	5 (20.8)	6 (26.1)	5 (21.7)	

BMI: Body Mass Index; PAL: Physical Activity Level. ^a^ Mean and standard deviation; ^b^ Median and interquartile range. * ANOVA trend; ** Kruskal–Wallis; ^#^ Chi-square. *p*-value highlighted in bold is significant.

**Table 2:** Comparison of the Brazilian Healthy Eating Index—Revised (BHEI-R), both total and components, and oxidative stress parameters of women between the baseline period (p0) and post adjuvant treatment for breast cancer (*n* = 70), Florianopolis City (SC), 2006–2011.

	p0	p1	*p*
Total BHEI-R ^b^	76.23 (71.21–83.17)	76.47 (73.01–81.21)	0.684 *
Total Fruits (0–5) ^b^	4.98 (3.68–5)	5 (3.16–5)	0.325 *
Whole Fruits (0–5) ^b^	5 (4.49–5)	5 (5–5)	**0.042** *
Total Vegetables (0–5) ^b^	4.92 (2.93–5)	3.53 (2.34–5)	**0.042** *
Dark Green and Orange Vegetables and Legumes (0–5) ^b^	5 (4.51–5)	5 (3.63–5)	0.084 *
Total Grains (0–5) ^b^	5 (5–5)	5 (5–5)	0.718 *
Whole Grains (0–5) ^b^	0 (0–0.51)	0 (0–0.52)	0.718 *
Milk and dairy products (0–10) ^a^	5.05 (0.34)	4.11 (0.27)	0.005 **
Meat, eggs and legumes (0–10) ^b^	8.8. (6.83–10)	6.79 (4.72–10)	<0.001 *
Oils (0–10) ^b^	10 (10–10)	10 (10–10)	0.421 *
Saturated Fat (0–10) ^b^	6.9 (4.33–9.06)	8.75 (7–10)	**<0.001** *
Sodium (0–10) ^b^	8.73 (7.05–9.67)	9.81 (8.58–10)	**<0.001** *
Calories from SoFAAS (0–20) ^b^	17.75 (13.52–20)	17.75 (14.21–20)	0.678 *
TBARS ^b^	4.81 (4.13–5.68)	8.69 (4.3–13.16)	**<0.001** *
LH ^b^	3.81 (2.84–6.06)	5.59 (1.80–9.75)	**0.046** *
Carbonyl ^b^	0.74 (0.57–1.14)	0.94 (0.86–1;07)	**0.023** *
GSH ^b^	76.02 (64.62–91.72)	79.65 (56.58–92.29)	0.932 *
FRAP ^a^	629.47 (19.09)	573.75 (22.66)	0.051 **

p0: baseline period; p1: post-treatment period. TBARS: Thiobarbituric acid-reactive substances; LH: Lipid hydroperoxide; GSH: Reduced glutathione; FRAP: Ferric reducing antioxidant power, Carbonyl: carbonyl protein. ^a^ Mean and standard deviation. ^b^ Median and interquartile range. * Wilcoxon. ** Paired t Test. *p*-value highlighted in bold is significant.

**Table 3:** Diet quality according to Brazilian Healthy Eating Index—Revised (BHEI-R) tertiles and its association with oxidative stress parameters of women with breast cancer at baseline (p0) (*n* = 70), Florianopolis City (SC), 2006–2011.

	Oxidative Stress Biomarkers														
BHEI-R	TBARS-log *			LH-log ^†^			Carbonyl-log ^‡^			GSH ^§^			FRAP ^§^		
Tertiles	β	(CI95%)	*p*	β	(CI95%)	*p*	β	(CI95%)	*p*	β	(CI95%)	*p*	β	(CI95%)	*p*
1st tertile	Ref			Ref			Ref			Ref			Ref		
2nd tertile	0.076	(−0.189; 0.341)	0.569	−0.098	(−0.544; 0.348)	0.661	−0.004	(−0.263; 0.255)	0.977	−8.296	(−20.947; 4.355)	0.195	70.083	(−17.512; 157.678)	0.115
3rd tertile	0.040	(−0.235; 0.314)	0.775	0.123	(−0.328; 0.575)	0.587	−0.270	(−0.542; 0.001)	0.051	−5.383	(−18.166; 7.399)	0.403	106.781	(18.273; 195.289)	**0.019**

p0: baseline period; CI95%: Confidence interval. TBARS: Thiobarbituric acid-reactive substances; LH: Lipid hydroperoxide; GSH: Reduced glutathione; FRAP: Ferric reducing antioxidant power, Carbonyl: carbonyl protein. * Adjusted by age. ^†^ Adjusted by alcohol, tumor stage and age. ^‡^ Adjusted by alcohol and age. ^§^ Adjusted by alcohol, schooling and age. *p*-value highlighted in bold is significant.

**Table 4:** Diet quality according to the Brazilian Healthy Eating Index—Revised (BHEI-R) of women with breast cancer and its association with changes in oxidative stress biomarkers between baseline period (p0) and post adjuvant treatment (p1) (*n* = 70) Florianopolis City (SC), 2006–2011.

BHEI-R	Increased Biomarkers									
	TBARS-log *		LH-log ^†^		Carbonyl-log ^‡^		GSH ^§^		FRAP ^ǁ^	
	OR (CI95%)	*p*	OR (CI95%)	*p*	OR (CI95%)	*p*	OR (CI95%)	*p*	OR (CI95%)	*p*
Reduction	1		1		1		1		1	
Increase	0.36	0.086	0.88	0.815	0.86	0.797	1.12	0.820	0.56	0.260
	(0.11–1.15)		(0.32–2.47)		(028–2.67)		(0.43–2.92)		(0.20–1.55)	

p0: baseline period; p1: post-treatment period. OR: Odds ratio; 95% CI: 95% Confidence interval. TBARS: Thiobarbituric acid-reactive substances; LH: Lipid hydroperoxide; GSH: Reduced glutathione; FRAP: Ferric reducing antioxidant power, Carbonyl: carbonyl protein. * Adjusted by surgery type, difference in waist circumference and age. ^†^ Adjusted by tumor stage and age. ^‡^ Adjusted by surgery type and age. ^§^ Adjusted by alcohol and age. ^ǁ^ Adjusted by difference in waist circumference and age.

## Data Availability

The data presented in this study are available on request from the corresponding author. The data are not publicy available due to privacy.
